# Feline lungworms unlock a novel mode of parasite transmission

**DOI:** 10.1038/srep13105

**Published:** 2015-08-14

**Authors:** Vito Colella, Alessio Giannelli, Emanuele Brianti, Rafael Antonio Nascimento Ramos, Cinzia Cantacessi, Filipe Dantas-Torres, Domenico Otranto

**Affiliations:** 1 Department of Veterinary Medicine, University of Bari, 70010 Valenzano, Bari, Italy; 2 Department of Veterinary Sciences, University of Messina, Messina 98122, Italy; 3 Department of Veterinary Medicine, University of Cambridge, Cambridge CB3 0ES, United Kingdom; 4 Department of Immunology, Aggeu Magalhães Research Centre, Oswaldo Cruz Foundation, 50670-420 Recife, Pernambuco, Brazil

## Abstract

Snail-borne lungworms exert an enormous toll on the health and welfare of animals and humans. Of these parasites, *Aelurostrongylus abstrusus* and *Troglostrongylus brevior* affect the respiratory tract of felids. These lungworms share both the ecological niche and the species of snail (*Helix aspersa*) acting as intermediate host. Recently, the ability of *H. aspersa* to shed infective third-stage larvae (L3s) of *A. abstrusus* and *T. brevior* in the environment has been demonstrated, matching previous knowledge of mode of transmission of zoonotic lungworms. Here, we evaluated, for the first time, the ability of *A. abstrusus* and *T. brevior* L3s to infect new, susceptible snail hosts following their release from experimentally infected molluscs, and refer to this novel route of parasite transmission as *intermediesis.* The implications of snail-to-snail transmission in the epidemiology of snail-borne diseases are also discussed.

Gastropod-borne diseases exert an enormous socio-economic impact on human and animal populations^1^. Based on recent estimates, >300 million people are affected by a wide range of snail-borne infections (e.g., schistosomiasis and opisthorchiasis), generally considered under the “neglected tropical diseases” umbrella (NTDs)^2^. Driven by global efforts to develop novel and effective strategies to control human schistosomiasis, snail-borne diseases have attracted the interest of the scientific community on a global scale^1^,^2^,^3^. Amongst snail-borne helminths with zoonotic potential, *Angiostrongylus cantonensis* (Strongylida, Angiostrongylidae) has been increasingly reported in humans^4^,^5^,^6^ as a frequent cause of eosinophilic meningitis^6^,^7^,^8^,^9^,^10^. The life cycle of *A. cantonensis* involves rats as definitive hosts, snails and slugs as intermediate hosts and various paratenic hosts (e.g., crabs, freshwater shrimps)^11^,^12^. Humans become infected through the consumption of molluscs, paratenic hosts or food contaminated by snail secretions containing the infective third-stage larvae (L3s)^13^,^14^, with the latter transmission route generally considered less likely to cause disease^14^. The importation of infected snails (e.g., *Achatina fulica*) in areas previously considered infection-free facilitates the spread of the parasite and thus plays an important role in the epidemiology of the disease^15^. In addition, *A. cantonensis* has been detected in lemurs, opossums, tamarins, falcons and non-human primates, in which it causes neurological disorders, and that led to speculations that this parasite may endanger wildlife species in areas where the infection is uncontrolled^16^,^17^,^18^,^19^. However, information on the fundamental biology and epidemiology of these snail-borne nematodes is scant, mainly because of intrinsic difficulties in collecting human data and the risks associated with working with pathogenic organisms. Studies of non-zoonotic nematodes, biologically and phylogenetically closely related to *A. cantonensis*, may allow us to overcome these obstacles and assist research aimed at shedding light on the effective role/s of snail intermediate hosts in the epidemiology of these diseases. For example, snail-borne helminths parasitizing the respiratory tract of cats (=feline lungworms) are well known in veterinary medicine because of the disease they cause in infected animals^20^. In particular, *Aelurostrongylus abstrusus* (Strongylida, Angiostrongylidae) is found in the bronchioles and alveolar ducts of the feline definitive host, whereas *Troglostrongylus brevior* (Strongylida, Crenosomatidae) is usually detected in the bronchi and bronchioles^21^. The adult nematodes live in the respiratory tract where they release first-stage larvae (L1s), which are excreted with the host faeces and penetrate suitable snail intermediate hosts, where they develop to L3s^22^. These nematodes share both the ecological niche and the gastropod species acting as intermediate host (e.g., *Helix aspersa*)^23^; very recently, our group has demonstrated the ability of infected *H. aspersa* to excrete infective L3s of both lungworms in the environment *via* the mucus or following death of the snail^24^. However, despite this evidence, very little is known of the biology of these parasites in their snail intermediate hosts. Elucidating fundamental aspects of snail-parasite interactions will not only lead to a better understanding of the epidemiology and transmission patterns of these and other (phylogenetically related) species, but will also assist the identification and development of novel control strategies focussed on the snail intermediate hosts. In particular, given that *A. cantonensis*, *A. abstrusus* and *T. brevior* share many taxonomic, systematic and biological features, comparative investigations of the epidemiology, ecology, biology and snail-parasite relationships of human and feline lungworms may provide useful clues towards the development of novel and effective treatment and control strategies against this group of parasites, in line with the principles of the One Health Initiative (http://www.onehealthinitiative.com). Therefore, in this study we (i) assess the potential of nematode transmission from infected to naïve susceptible snails; and (ii) evaluate the survival time of *A. abstrusus* and *T. brevior* L3s in the environment.

## Results

In order to assess the potential of L3s to infect naïve intermediate hosts, 24 pathogen-free *H. aspersa* snails were each experimentally infected with 50 L3s of either *A. abstrusus* or *T. brevior*. Larvae of *A. abstrusus* were 540 μm long and 27 μm wide, whereas larvae of *T. brevior* measured 435 μm in length and 21 μm in width, on average. L3s were recovered without the external sheaths, they were motile and identified as infective L3s based on a previous description^25^. The numbers of *A. abstrusus* and *T. brevior* larvae subsequently detected in the foot and viscera of *H. aspersa* are reported in Table 1. Out of 168 *A. abstrusus* larvae retrieved (mean 21 ± 12.1), 78 (46.4%) were localized in the foot and 90 (53.6%) in the viscera. Given the initial infection dose, from 12 to 66% L3s were retrieved, with a number of live larvae significantly larger than that of the dead (p ≤ 0.05). Only five larvae of *T. brevior* were recovered from the viscera (n = 4) and the foot (n = 1) and, of these, only one was alive (Table 1).

**Figure 1:**
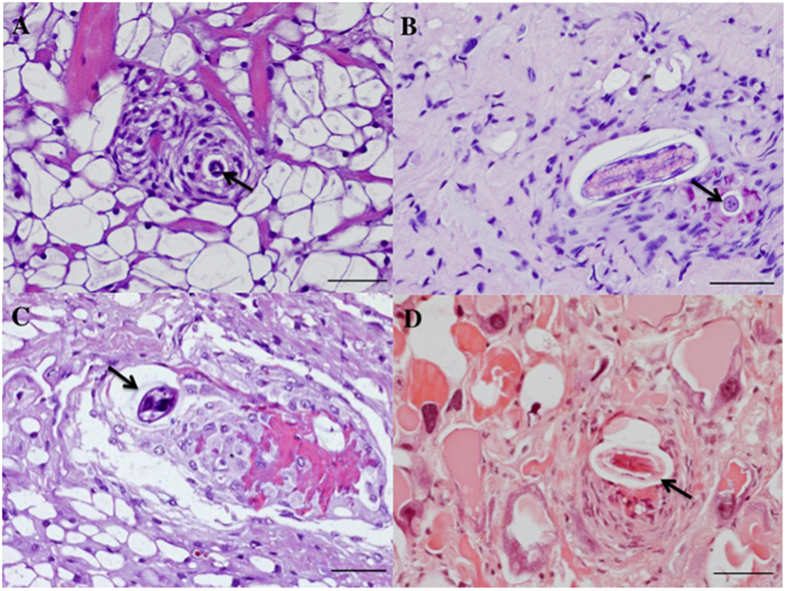
Histopathology of snail tissue. Third-stage larvae of *Aelurostrongylus abstrusus* from experimentally infected gastropods were detected in the fibro-muscular tissue of the foot (**1A,B**), and in glands (**1C,D**). Scale bar 50 μm.

Histologically, L3s of *A. abstrusus* were detected at all timepoints in the eight experimentally infected snails. Conversely, no larvae of *T. brevior* were observed at the histological examination of gastropods, which was in accordance with the small number of larvae detected at the artificial digestion (n = 1 per snail). L3s of *A. abstrusus* were detected in the fibro-muscular tissue of the foot (Fig. 1a,b) and in the intestine, kidney parenchyma, and locomotion-associated glands (Fig. 1c,d).

Then, in order to evaluate the occurrence of snail-to-snail transmission of L3s, molluscs infected by either *A. abstrusus* or *T. brevior* were co-housed with uninfected snails (study 1). Live L3s of *A. abstrusus* (n = 5) were detected in previously uninfected snails at 8 and 12 days post contact (dpc) and only one of *T. brevior* at 1 dpc. Subsequently, one dead snail infected by either lungworm species was co-housed with susceptible uninfected snails (study 2). Live L3s of *A. abstrusus* (n = 3) were then detected in uninfected snails at 1, 8 and 12 dpc, whereas those of *T. brevior* were dead and detected at 1 (n = 3) and 12 dpc (n = 1), respectively.

### Survival of third-stage larvae

**Figure 2:**
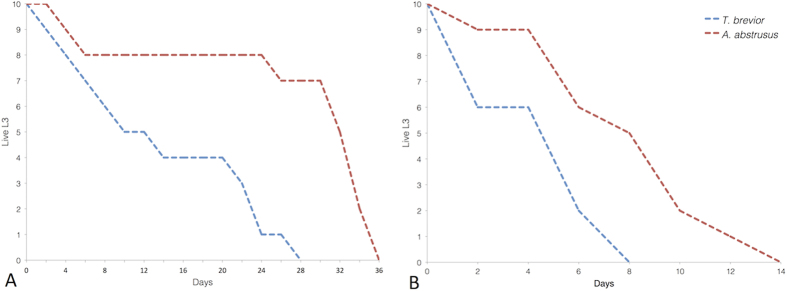
Number of live third-stage larvae of *Aelurostrongylus abstrusus* and *Troglostrongylus brevior* at 4 °C (A) and 26 °C (B).

Finally, we evaluated the off-host survival time of *A. abstrusus* and *T. brevior* L3s at different temperatures *via* observation at 2 day-intervals. The maximum survival time of L3s at 4 and 26 °C was 36 and 14 days for *A. abstrusus* and 28 and 8 days for *T. brevior* (Fig. 2).

For both parasites, an inverse association (p > 0.0001) between the timepoint and the number of live larvae was registered at both temperatures. At 4 °C, the frequency of *T. brevior* live larvae was higher (p ≤ 0.05) than that of dead larvae until the eighth day; at 26 °C, the same trend was observed, but only on the fourth day. For *A. abstrusus*, a larger number of live larvae (p ≤ 0.05) was recorded until the thirty-second and sixth day at 4 and 26 °C, respectively (Fig. 2).

Molecular characterisation of larvae recovered from the experimental studies, based on amplification and sequencing of the internal transcribed spacer 2 of the ribosomal DNA (ITS-2) confirmed the morphological identification, with all sequences displaying 100% identity to those of *A. abstrusus* and *T. brevior* in GenBank^TM^ (accession numbers KF751655 and KF751656, respectively).

## Discussion

In this article we report, for the first time, snail-to-snail transmission of *A. abstrusus* L3s from infected to naïve *H. aspersa*. While the transmission of infective larvae through a series of paratenic hosts, also known as paratenesis, is a well-documented mechanism occurring in selected parasites (e.g., *Gnathostoma spinigerum*)^26^, the transmission of L3s between intermediate hosts is, to the best of our knowledge, a completely novel finding. More in general, this is the first report of an intermediate host being infected by an infective L3. Such an occurrence may have important implications for the epidemiology of a number of snail-borne parasitic diseases (e.g., *A. cantonensis*, *Protostrongylus* spp.), whose L3s may be excreted by snails^13^,^27^,^28^. However, the impact of this transmission route on the overall patterns of parasite epidemiology and ecology (i.e. compared to the ingestion of intermediate or paratenic hosts)^29^ is currently unknown (given that the infection of a receptive host with L3s was not possible for ethical reasons) and deserves further consideration. Indeed, although the risk of human infection by *A. cantonensis* L3s excreted via snail mucus or contaminated water has been considered less likely to cause disease^14^, snail-to-snail transmission might increase the chance for the definitive host to come across the parasite. At the same time, this phenomenon may significantly increase the survival time of infective larvae in the environment since, once released by an intermediate host, they can promptly infect a new, susceptible snail should a suitable definitive host be unavailable. This hypothesis is further corroborated by the high recovery rate of *A. abstrusus* L3s (i.e., up to 66%) in experimentally infected *H. aspersa* and by the larval transmission from infected to uninfected snails (study 1). It is known that the mucus of snail trails is a source of energy intake when ingested by congeners^30^,^31^ and it is pivotal for aggregation and mating^31^. Snail clustering and the elimination of *A. abstrusus* L3s *via* the mucus may promote the circulation of the parasite among intermediate hosts, as supported also by the detection of infective larvae in the viscera of snails. Whether these metastrongyloids activate (directly or indirectly) a cascade of biochemical signals into the snail mucus, similarly to the sea snail *Littorina littorea* when infected by trematode parasites^32^, is yet to be confirmed.

Previous studies have shown that L3s of *A. abstrusus*, *T. brevior* and *Muellerius capillaris* leave the infected snails soon after the death of their intermediate hosts^24^,^33^. This knowledge supports our finding of L3s in previously uninfected snails following direct contact with a dead infected specimen (study 2). Compared to *A. abstrusus*, L3s of *T. brevior* displayed a decreased capacity to infect *H. aspersa*, a possible consequence of a limited adaptation of the latter species to this snail intermediate host. These data are supported by previous evidence of a higher moulting rate for *A. abstrusus* than *T. brevior* in the same snail species^24^.

The passage of infective larval stages between intermediate hosts is a novel pattern of parasite transmission. Therefore, we propose for this mode of transmission the term *intermediesis*.

One of the primary effects of the circulation of infective larvae among snails is the broadening of the number of intermediate hosts available to the definitive hosts, which may ultimately lead to the spread of the infection in a suitable environment. This mechanism allows the enlargement of *refugia* of infective larvae whose survival time is a limiting factor in hostile environments (i.e., high temperatures, low rainfall or other abiotic factors), thus persisting from season to season across time and space. For example, the effect of human activities (e.g., irrigation or water storage) has been hypothesized to play a major role in the spread of snail-borne diseases. However, the elucidation of snail-parasite relationships and of new transmission pathways is central to the development of integrated control strategies^2^, that aim at overcoming concerns related to drug resistance^34^,^35^,^36^. If this transmission pattern is confirmed to occur in nature, mathematical models will need to integrate the phenomenon of “intermediesis”, besides paratenesis, in studies of transmission dynamics of parasites with a complex life cycle^37^.

## Methods

### Experimental infection of gastropods by L3s

Infective L3s of *A. abstrusus* or *T. brevior* were obtained from infected *H. aspersa* snails maintained in the Parasitology Laboratory of the University of Bari (Puglia, Italy) as follows. Each snail was digested in 100 ml HCl solution (pH 2.2) and 3 mg/ml of pepsin (Sigma-Aldrich, St. Louis, Missouri, United States). The suspension was heated on a magnetic stirrer at 37 °C for 75 min, sift through a 250 μm sieve to remove undigested material, transferred to 50 ml plastic tubes and centrifuged at 600 *g* for 5 min. The suspension was microscopically examined and larvae were morphologically identified, according to previous descriptions^23^,^25^, with the aid of a computer software (Leica LAS^®^ AF 4.1). Single infective doses of 50 L3s were collected under a light microscope (Leica^®^, DM LB2) and kept in plastic tubes (1.5 ml), until being utilized for snail infection. Nematode-free snails, 20 months old, were purchased from a commercial provider in Barletta (Puglia, Italy), placed in plastic boxes (=*vivaria*) and fed every 2 days with lettuce and water. The *vivaria* were kept in a temperature-controlled room (23 ± 1 °C) and monitored via a thermo-hygrometer on an hourly basis. The absence of natural infections by any nematode larvae was assessed by microscopically examining eight snails (10%) the day prior to the infection. On the day of the infection, two groups of 12 (nematode-free) snails each were infected with 50 live L3s of either *A. abstrusus* or *T. brevior*. Briefly, snails were individually placed in a plastic infection chamber containing a potato slice (0.5 cm thick) and the infective dose of either *A. abstrusus* or *T. brevior*. Specimens were left in the infection chamber for 48 h and subsequently returned to the *vivaria*. Successful infection with either metastrongyloid was assessed at different time points (i.e., 1, 4, 8, 12 days post-infection), using the protocol described above on two sections of each snail, i.e. muscular foot and viscera.

The presence of larvae from experimentally infected gastropods was detected by histological examination. At each time point, one snail infected by L3s of *A. abstrusus* or *T. brevior* was examined. Snails were anesthetized with menthol steam, deprived of their shells and fixed in a 50 mL vial with 10% neutral buffered formalin solution. Sections of 5 μm across the body of the snail were stained with haematoxylin and eosin (H&E) and routinely processed.

### Snail to snail transmission

Two studies were designed to determine the potential of snail-to-snail transmission of infective L3s from infected to naïve, susceptible gastropods. In study 1, infected snails (n = 3) marked with permanent ink were co-housed with uninfected specimens (n = 3) in a 1 L container. The experiment was repeated twice (i.e., G1a and G1b) for each nematode species.

In study 2, for each species, one dead infected mollusc was co-housed soon after its natural death with six uninfected snails in a 1 L container for 24 h. All naïve snails from both studies were artificially digested at three time points (i.e., 1, 8, 12 days post-contact; dpc) for the detection of L3s and examined at each time point (i.e., one specimen per group in study 1 and two snails for study 2). At the end of the study, infected snails from study 1 and the dead specimen from study 2 were digested to confirm the success of the experimental infection.

### Survival of third-stage larvae

The survival time of *A. abstrusus* and *T. brevior* L3s was recorded at 4 and 26 °C, the minimum and maximum temperature at which snails are active. Briefly, following artificial digestion of *H. aspersa*, the solution was centrifuged at 600 *g* for 5 min, and the larvae-containing sediments were collected and washed, twice. Following the last centrifugation, the sediments were transferred to 1.5 ml tubes (one for each nematode) and maintained at 4 and 26 °C, respectively.

The motility of the nematode larvae (10 larvae per time-point) was assessed at 2-day intervals until the death of the last specimen (maximum survival time). Larvae were considered dead when no movements were observed under a light microscope (Leica^®^, DL MB2) for up to 10 s, or when degeneration of larval internal organs occurred.

Data were analysed by Fisher’s exact test using BioEstat software (version 5.0; Mamirauá/CNPq, Belém, PA, Brazil).

## Additional Information

**How to cite this article**: Colella, V. *et al.* Feline lungworms unlock a novel mode of parasite transmission. *Sci. Rep.* **5**, 13105; doi: 10.1038/srep13105 (2015).

## Figures and Tables

**Table 1:** Number of third-stage larvae of *Aelurostrongylus abstrusus* and *Troglostrongylus brevior* detected in the foot and viscera of *Helix aspersa* snails infected with 50 L3s each.

Lungworms	Dpi*	First specimen			Second specimen		
		Foot	Viscera	Total	Foot	Viscera	Total
*Aelurostrongylus abstrusus*	1	18 (1)	5 (1)	23 (2)	3	3	6
	4	5	4	9	13 (3)	29 (6)	42 (9)
	8	3	8 (1)	11 (1)	18 (2)	13	31 (2)
	12	7	15	22	11	13	24
*Troglostrongylus brevior*	1	—	1	1	—	1 (1)	1 (1)
	4	—	—	—	—	1 (1)	1 (1)
	8	1 (1)	—	1 (1)	—	1 (1)	1 (1)
	12	—	—	—	—	—	—

Two specimens were analysed at each time point (i.e., 1, 4, 8, 12 ^*^Days post infection, Dpi). Number of dead larvae in brackets.
